# Implementation of public health tasks by Polish municipalities in 2019–2024: a cross-sectional study

**DOI:** 10.3389/fpubh.2026.1932172

**Published:** 2026-09-10

**Authors:** Katarzyna Wnuk, Jakub Świtalski, Edyta Krzych-Fałta, Anna Augustynowicz

**Affiliations:** 1 School of Public Health, Centre of Postgraduate Medical Education of Warsaw, Warsaw, Poland; 2 Department of Health Policy Programs, Department of Healthcare Services, Agency for Health Technology Assessment and Tariff System, Warsaw, Poland; 3 Department of Ethics and Medical Law, Center for the Humanities and Social Sciences in Medicine, Medical University of Warsaw, Warsaw, Poland; 4 Department of Nursing Propedeutics, Faculty of Health Sciences, Medical University of Warsaw, Warsaw, Poland; 5 VIZJA University, Warsaw, Poland

**Keywords:** government programs, health policy, local government units, municipalities, Poland, public health, public health practice

## Abstract

**Background:**

Public authorities, including local government units, play a fundamental role in shaping public health (PH) policy. To date, the activities of self-government communities in the domain of PH have not been the subject of scholarly research. The study aims to characterise the activity of Polish municipalities in implementing PH tasks during the period 2019–2024.

**Materials and methods:**

The cross-sectional survey was conducted via Computer-Assisted Web Interview using a proprietary questionnaire prepared with input from experts in PH. All municipalities in Poland were invited to participate (*N* = 2,479). Statistical analysis was conducted using IBM SPSS Statistics 29, which included frequency analysis, Mann–Whitney U tests, Kruskal-Wallis tests, and cross-tabulations with a chi-square test of independence. Verification was conducted to determine whether the type of municipality, the province in which it is located, the number of employees in the municipal office responsible for PH issues, the operation of healthcare entities within the municipality, and the existence of a Municipal Development Strategy influence the implementation of PH tasks.

**Results:**

The analysis included responses from 601 municipalities, with 69.9% having performed PH tasks between 2019 and 2024. Rural municipalities perform PH tasks less frequently (*χ*^2^ = 17.20; *p* < 0.001), including prevention programmes, compared to urban municipalities. Municipalities that conducted PH tasks have statistically significantly more employees (Z = −6.80; *p* < 0.01) and hospitals (*χ*^2^ = 10.18; *p* = 0.001). Moreover, the presence of a Municipal Development Strategy during the analysed years was also significantly linked to the implementation of these tasks (*χ*^2^ = 10.16; *p* = 0.006). However, no statistically significant correlations were found when comparing different provinces.

**Conclusion:**

Adequate measures should be implemented to support the development of health policies in smaller local authorities. Factors that warrant particular attention are the number of employees involved in PH tasks, the number of potential task implementers, and the existence of a Municipality Development Strategy.

## 1 Background

In Polish legislation, the legal framework for the systemic organisation of public health (PH) is outlined in the Public Health Act (1). As outlined in Article 2, PH tasks encompass the following key areas: monitoring the health status of the population, assessing health risks and overall quality of life; providing health education addressed to various social groups, with a particular focus on children, youth, and the older adults; promoting health initiatives and implementing disease prevention strategies; identifying, eliminating, or mitigating threats and harm to both physical and mental health across various environments, including homes, schools, workplaces, and recreational settings; evaluating the relevance and effectiveness of healthcare services in relation to the health needs of the population; initiating and conducting scientific research and fostering international collaborations in PH; training personnel involved in implementing PH tasks; limiting health disparities resulting from socio-economic factors and encouraging public engagement in health matters. A convergence can be observed between the tasks defined in Article 2 of the Public Health Act and the PH functions outlined by the World Health Organization (WHO) for the European Region (2).

International evidence demonstrates that local governments play an important role in planning, implementing, and supporting PH interventions. Reported activities encompass vaccination campaigns, obesity prevention programmes, health education initiatives, promotion of physical activity and healthy nutrition, chronic disease prevention, and actions addressing broader determinants of health. In many settings, these interventions are delivered through collaboration between local authorities, healthcare providers, and community organisations. The range of activities identified in the literature corresponds to those undertaken by local government units in Poland and reflects the core PH functions defined by the WHO (3–6).

Implementing core PH functions is the domain of public authorities. It requires collaboration and cross-sectoral initiatives at all levels of government, at every stage of organising PH activities, as well as the involvement of various entities, including non-governmental organizations (NGOs) and local communities. Institutions implementing these functions may vary in organisational structure depending on the specific health activities in each country (7, 8). According to this legislation, the implementation of PH duties is a collaborative effort involving governmental administrative bodies, state organisational units, including executive agencies and local government units at all levels, each tasked with promoting and protecting health (municipalities, counties, and provincial governments).

In Poland, there are three levels of local government units: self-governments of the voivodeships (16 administrative regions in Poland); poviats, which cover part of the voivodeship (314 poviats and 66 cities with poviat rights); basic units of local government, called communes (302 urban, 718 urban–rural and 1,459 rural communes) (9). The type of municipality depends, among other factors, on the population size and its legal status. A rural municipality consists solely of villages. An urban–rural municipality typically comprises a town and its surrounding villages. An urban municipality, on the other hand, includes only the city (10, 11).

Local self-government communities, specifically municipalities, should play a crucial role in implementing PH initiatives. These communities are best positioned to identify the health needs of their residents. Additionally, they possess knowledge of specific local conditions, which can be very important in selecting the most effective actions, including motivating participation in health initiatives. To implement local policy more effectively, local governments prepare a so-called Municipal Development Strategy, which defines, among other things, strategic development objectives and directions for action in social, economic, spatial, and climate-environmental dimensions (1). One of the areas covered by this strategy may also be health protection.

To date, there has been no scientific research on the activities of Polish municipalities in implementing PH tasks. However, in line with the Public Health Act (1), all local government units in Poland are required to report annually to the provincial governor by 31 March on the PH tasks they implemented or that are currently ongoing from the previous year. Reports can be submitted in writing or electronically, using the provided template and specifying the required information, including the type, duration, and population covered, as outlined in the Minister of Health Regulation (12).

After verifying whether these activities align with the objectives of the National Health Programme, the provincial governor prepares a summary report with an opinion and submits it to the Minister of Health by 30 September. However, the factors affecting the implementation of these tasks or obstructing initiatives in this area are not identified. Recognising these factors could enhance the effectiveness and efficiency of health-related measures, and consequently local health policies aimed at improving the health status of the municipal population.

This study seeks to characterise municipal activities in the execution of PH tasks between 2019 and 2024. It examines the differences between types of municipalities in terms of the extent of task implementation, the number of municipal employees handling PH issues, and healthcare entities operating within the municipality. Additionally, it verifies whether the existence of a Municipal Development Strategy affects the implementation of PH tasks.

This article is the first in a series of research papers examining the activities of municipalities in implementing PH tasks. Future papers will shed light on topics such as financing, fundraising and the determinants of PH planning, implementation, and delivery.

## 2 Materials and methods

### 2.1 Study design

The cross-sectional study was conducted utilising the Computer-Assisted Web Interview (CAWI) method, employing a proprietary survey questionnaire. The survey targeted all local government units at the municipal level. Data on municipalities was sourced from the contact details database of local government units provided by the Ministry of the Interior and Administration (13).

On 25 January 2025, all municipalities in Poland (*N* = 2,479) received an e-mail containing a cover letter requesting their participation in the survey, along with a link to the survey questionnaire. To enhance respondent engagement, randomly selected municipalities were contacted via telephone, and an e-mail reminder concerning the ongoing survey was disseminated on 16 February 2025. The collection of completed questionnaires concluded on 28 February 2025.

### 2.2 Questionnaires used

The development of a reliable and accurate research tool unfolded in several stages. Initially, the principal investigator created a proprietary survey questionnaire comprising 18 thoughtfully designed sets of questions. Following this, an expert panel was appointed, which included the National Consultant in Public Health, the Mazovia Province Consultant specialising in PH, representatives from the Department of Public Health and the Legal Department of the Ministry of Health, as well as a representative from the National Institute of Public Health – National Institute of Hygiene – National Research Institute (National Institute of Public Health NIH – NRI).

Over the course of three meetings, experts assessed the content of the prepared questions and suggested modifications to the questionnaire. These amendments were mainly linguistic, aimed at clarifying the content of the questions. Furthermore, a decision was reached to remove two questions that, in the experts’ opinion, did not meet the substantive criteria. Consequently, the questionnaire ultimately consisted of 16 questions.

Subsequently, to verify the correctness and feasibility of the survey, a pilot study was conducted in November 2024 across three municipalities of each type. The feedback received pertained solely to three questions, indicating the necessity for linguistic modifications and additional explanations to facilitate the completion process of the questionnaire. Consequently, after considering the comments from municipal representatives, the final version of the survey questionnaire was prepared in electronic format utilising the EUSurvey system, which serves as an online survey generator (14).

The survey questionnaire consists of 4 sections. The first section includes a field designated for entering the municipality identifier, in accordance with the National Official Register of Territorial Division of the Country (TERYT). The TERYT register is a classification and registration system for territorial divisions in Poland, operating under the relevant legal regulations that regulate the management and updating of territorial data. Numerical symbols are assigned to each municipality to enable the identification of its type and category (15, 16).

Section two comprises five questions designed to gather general information about the management and social infrastructure of the respective municipality. The subsequent eight questions delve into aspects of PH, focusing on topics such as securing funding for related activities and the challenges faced in planning, implementing, executing and reporting PH initiatives. The final section comprises two questions, along with one open-ended query. One of the questions addresses issues related to the implementation of disease prevention programmes (including vaccination, screening, and addiction prevention programmes). Conversely, the second question utilises a 5-point Likert scale (ranging from 1 – strongly disagree to 5 – strongly agree), allowing respondents to share their views on key elements pertinent to PH tasks (among others, legal frameworks, funding, and the implementation process). The last question (optional) provides space for any additional comments on the implementation of PH activities or the survey tool.

In the instructions and description section of the research tool, each participant was informed about the study’s objectives and assured that their provided TERYT code would be kept anonymous.

The data acquired covers the period from 2019 to 2024, a timeframe chosen in light of the COVID-19 pandemic. In the initial years of the pandemic (2020–2022), local governments’ activities regarding PH were primarily focused on efforts aimed at mitigating the pandemic’s impact. To this end, we decided to include data from the year preceding the pandemic as well as the years following it to minimise the influence of COVID-19 on our findings. Additionally, the chosen timeframe aligns with the current term of local authorities, which was extended to 2024 due to the effects of the pandemic (17).

The complete survey questionnaire is available in the Supplementary material.

### 2.3 Statistical analysis

Statistical analysis was conducted using IBM SPSS Statistics 29, which included frequency analysis, the Mann–Whitney U test, the Kruskal-Wallis ANOVA test, and cross-tabulation with the chi-square independence test (*χ*^2^). The significance level was set at *α* = 0.05.

For crosstabs larger than 2×2, the significance of the deviation of the actual results from the expected results was evaluated based on the standardised residual values, using a reference point of |1.96| residual values, using |1.96| as the reference point this approach stems from the distribution of standardised values Z and helps determine which observed values deviate from expected values at a significance level of *p* < 0.05. Consequently, it was possible to identify which responses occurred with greater or lesser frequency than would be anticipated under the assumption of no dependence.

For each test, effect size indices were calculated: *χ*^2^ (0.1 – weak; 0.3 - moderate; 0.5 - strong; *ϕ* was calculated for 2×2 tables, while Vc was calculated for tables larger than 2×2), Mann–Whitney U (*r*: 0.1 - weak; 0.3 - moderate; 0.5 - strong) and Kruskal–Wallis (*η*^2^: 0.01 - weak; 0.06 - moderate; 0.14 - strong) (18, 19).

As part of the overall characterisation of municipalities, an analysis was performed on how often responses were given to subsequent survey questions.

To determine if the type of municipality or province was associated with specific aspects of PH task implementation, cross-tabulation tables were generated, and the chi-square test of independence was used for nominal variables. For ordinal variables, the Kruskal-Wallis ANOVA test was employed.

Furthermore, to assess differences in the implementation of PH tasks based on the characteristics of municipal management and infrastructure, cross-tabulation tables were created using the chi-square test of independence or the Mann–Whitney U test. To identify which results differed statistically significantly, post-hoc tests (specifically, Dunn-Bonferroni pairwise comparison tests) were conducted.

### 2.4 Ethical considerations

This study was conducted in accordance with the principles outlined in the Declaration of Helsinki and received approval from the Bioethics Committee at the Medical University of Warsaw, confirming its adherence to the standards of scientific research ethics (AKBE/49/2025).

## 3 Results

### 3.1 Sample characteristics

By 28.02.2025, a total of 629 completed questionnaires had been received (response rate: 25.4%). After creating the target database and verifying response accuracy, 28 questionnaires were excluded because they contained missing or incorrect TERYT municipality identifiers or had multiple responses from the same municipality. Ultimately, the survey comprised responses from 601 municipalities across 16 provinces, including 74 urban (12.3%), 370 rural (61.6%), and 157 urban–rural municipalities (26.1%). The study group encompassed 23.5% of urban, 25.4% of rural, and 21.9% of urban–rural municipalities nationwide.

The most represented provinces were Mazovia (*n* = 62; 10.3%) and Lublin (*n* = 59; 9.8%). Conversely, the fewest questionnaires were received from the Opole province (*n* = 14; 2.3%). Detailed information on the distribution of responses in individual provinces is presented in the Supplementary Table S1.

Municipalities most often had one person responsible for addressing PH issues (73.9%). In contrast, in 11.8% of municipalities, this subject was managed by two employees, and in 10.5% of cases, by none of the employees. The remaining municipalities (3.8%) indicated ≥ 3 employees.

Most municipalities did not have a functioning hospital (83.2%; *N* = 500). In terms of the number of entities providing primary healthcare (PHC), most municipalities had one such entity (approximately 38%–39% of municipalities), followed by two entities (approximately 21%–22% of municipalities) or three entities (approximately 9%–10% of municipalities). A larger number of entities appeared sporadically. Table 1 presents information on the number of primary healthcare providers (PHC) in the analysed period.

**Table 1:** Number of primary health care (PHC) providers operating in municipalities in 2019–2024.

Number of PHC providers	2019		2020		2021		2022		2023		2024	
	*n*	%	*n*	%	*n*	%	*n*	%	*n*	%	*n*	%
0	18	3.0	19	3.2	19	3.2	19	3.2	20	3.3	20	3.3
1	236	39.3	234	38.9	231	38.4	234	38.9	231	38.4	233	38.8
2	129	21.5	130	21.6	133	22.1	130	21.6	134	22.3	135	22.5
3	55	9.2	57	9.5	59	9.8	62	10.3	61	10.1	58	9.7
4	21	3.5	21	3.5	23	3.8	21	3.5	21	3.5	20	3.3
5	12	2.0	12	2.0	10	1.7	11	1.8	10	1.7	10	1.7
>5	23	3.8	22	3.7	21	3.5	22	3.7	23	3.8	26	4.3
No data*	107	17.8	106	17.6	105	17.5	102	17.0	101	16.8	99	16.5

*Optional question; therefore, there were gaps in the data for responses on the number of PHC providers.

The primary initiators of PH tasks were heads of municipalities/mayors/city mayors (64.8%), followed by healthcare units (8.3%) and county self-government (5.5%). Detailed information on the initiators, as indicated by municipalities, is available in the Supplementary Table S2.

Approximately 55.9% of municipalities reported having a Municipal Development Strategy (MDS) for the period 2019–2024, while around 25.1% had one covering part of that period. Conversely, 18.8% lacked such a strategy, and one municipality did not answer this question (0.2%). Among the 487 municipalities with an MDS, 66.1% included objectives related to PH tasks.

### 3.2 Implementation of public health tasks by type of local government

From 2019 to 2024, 69.9% of municipalities (*n* = 420) reported performing PH tasks, while the remaining 30.1% (*n* = 181) indicated that they did not carry out any such tasks.

The analysis revealed a statistically significant relationship between the type of municipality and the implementation of PH tasks (*χ*^2^ = 17.20; *p* < 0.001; Vc = 0.17). It was noted that urban municipalities more frequently (86.5%) implemented PH tasks, whereas rural municipalities did so less often (64.3%). In contrast, no statistically significant differences were found among urban–rural municipalities (Table 2).

**Table 2:** Implementation of public health tasks by type of municipality in 2019–2024.

Implementation of public health tasks		Urban	Rural	Urban–rural	Total	*χ*^2^ (2)	*p*	Vc
Yes	*N*	64	238	118	420	17.20	<0.001	0.17
	%	86.5	64.3	75.2	69.9			
	Residual	3.30	−3.80	1.70				
No	*N*	10	132	39	181			
	%	13.5	35.7	24.8	30.1			
	Residual	−3.30	3.80	−1.70				
Total	*N*	74	370	157	601			
	%	100	100	100	100			

N – sample; *χ*^2^ – chi-square test result; *p* – statistical significance; Vc – effect size index (V Cramer).

A statistically significant correlation was observed between municipality type and the implementation of tasks related to protective vaccinations (*χ*^2^ = 11.69; *p* = 0.003; Vc = 0.17), screening tests (*χ*^2^ = 7.50; *p* = 0.024; Vc = 0.15), and addiction prevention (*χ*^2^ = 8.71; *p* = 0.013; Vc = 0.15) (Table 3).

**Table 3:** Implementation of disease prevention programmes in the scope of protective vaccinations, screening tests, and addiction prevention by municipality type in 2019–2024*.

Implementation of disease prevention tasks			Urban	Rural	Urban–rural	Total	*χ*^2^ (2)	*p*	Vc
Preventive vaccinations	Yes	*N*	36	106	58	200	11.69	0.003	0.17
		%	64.3	44.0	59.8	50.8			
		Residual	2.20	−3.40	2.00				
	No	*N*	20	135	39	194			
		%	35.7	56.0	40.2	49.2			
		Residual	−2.20	3.40	−2.00				
	Total	*N*	56	241	97	394			
		%	100	100	100	100			
Screening tests	Yes	*N*	20	65	38	123	7.50	0.024	0.15
		%	44.4	29.8	44.2	35.2			
		Residual	1.40	−2.70	2.00				
	No	*N*	25	153	48	226			
		%	55.6	70.2	55.8	64.8			
		Residual	−1.40	2.70	−2.00				
	Total	*N*	45	218	86	349			
		%	100.0	100	100	100			
Addiction prevention	Yes	*N*	50	186	85	321	8.71	0.013	0.15
		%	94.3	78.8	86.7	82.9			
		Residual	2.40	−2.70	1.20				
	No	*N*	3	50	13	66			
		%	5.7	21.2	13.3	17.1			
		Residual	−2.40	2.70	−1.20				
	Total	*N*	53	236	98	387			
		%	100	100	100	100			

*Optional question; therefore, there were inconsistencies in the other answers to this question. As a result, during the verification of correct answers, municipalities that reported not carrying out tasks in a specific area were excluded from this analysis, despite indicating in other parts of the question that they engaged in such activities.

N – sample; *χ*^2^ – chi-square test result; *p* – statistical significance; Vc – effect size index (V Cramer).

Protective vaccinations were more frequently implemented in urban (64.3%) and urban–rural (59.8%) municipalities, and less often in rural municipalities (44.0%). Similarly, screening activities were more common in urban–rural municipalities (44.2%) and less in rural ones (29.8%). Conversely, addiction prevention initiatives were more often implemented in urban municipalities (94.3%) compared to rural municipalities (78.8%). Detailed results are provided in Table 3.

There was no statistically significant correlation between the type of province and the implementation of PH tasks (*χ*^2^ = 24.40; *p* = 0.059; Vc = 0.20). The proportion of municipalities carrying out PH tasks was comparable across various provinces. Detailed information on the implementation of PH tasks in the provinces is presented in Supplementary Table S3.

### 3.3 Implementation of public health tasks based on the number of staff engaged in public health issues

The test results indicated statistically significant differences between municipalities in terms of the number of employees dealing with PH issues (H = 38.60; *p* < 0.001; *η*^2^ = 0.06) (Table 4). *Post-hoc* tests were conducted to determine statistically significant differences among the results. The analysis indicated that urban municipalities employed significantly more individuals than rural municipalities (*p* < 0.001) and urban–rural municipalities (*p* < 0.001). In turn, there were no significant differences (*p* = 0.226) between rural municipalities and urban–rural municipalities in this regard.

**Table 4:** Number of employees engaged in public health issues by type of municipality in 2019–2024.

Number of municipal employees	Mean rank	*Me*	*IQR*	*H* (2)	*p*	*η* ^2^
Urban (*n* = 74)	387.72_a, b_	2.00	1.00	38.60	<0.001	0.06
Rural (*n* = 370)	282.07_a_	2.00	0.00			
Urban–rural (*n* = 157)	304.74_b_	2.00	0.00			

The averages dividing the letter index differ from each other at the level of *p* < 0.05 (Dunn-Bonferroni test).

*n* – sample; *Me* – median; IQR – interquartile range; *H* – result of the Kruskal-Wallis test; *p* – statistical significance; *η*^2^ – effect size index (eta squared).

Regarding the number of employees handling PH issues, no significant differences were observed between provinces (H = 19.17; *p* = 0.206; *η*^2^ = 0.01). Consequently, the number of staff involved in social inclusion matters was consistent across regions. Further details on employee numbers by province are available in the Supplementary Table S4.

The analysis revealed significant differences in the number of employees managing PH tasks based on whether the municipality implemented tasks in the area (Z = −6.80; *p* < 0.01; *r* = 0.28). Municipalities that implemented such tasks had a higher number of employees involved in PH issues (Supplementary Table S5).

### 3.4 Implementation of public health tasks in relation to the operation of healthcare entities

A statistically significant correlation was noted between the implementation of PH tasks and the functioning of hospitals (*χ*^2^ = 10.18; *p* = 0.001; *ϕ* = 0.13). In municipalities with hospitals, approximately 83.2% successfully carried out PH tasks, while in those without hospitals, the percentage was approximately 67.2% (Table 5). However, regarding primary healthcare (PHC), no significant differences were observed in the number of these entities between 2019 and 2024, depending on whether the municipality undertook PH tasks (Table 6). The count of PHC centres remained relatively consistent across subsequent years, irrespective of the municipality’s engagement in PH tasks.

**Table 5:** Implementation of public health tasks in relation to the operation of a hospital in the municipality in 2019–2024.

Implementation of public health tasks	Variable				Total		*χ*^2^ (1)	*p*	*ϕ*
	Hospital – yes		Hospital – no						
	*N*	%	*N*	%	*N*	%			
Yes	84	83.2	336	67.2	420	69.9	10.18	0.001	0.13
No	17	16.8	164	32.8	181	30.1			
Total	101	100	500	100	601	100			

*N* – sample; *χ*^2^ – chi-square test result; *p* – statistical significance; ϕ – effect size index.

**Table 6:** Implementation of public health tasks in relation to the number of primary health care providers in the period 2019–2024.

Variable	Yes				No				*Z*	*p*	*r*
Number of PHC providers	*n*	Mean rank	*Me*	*IQR*	*n*	Mean rank	*Me*	*IQR*			
2019	346	253.01	1.00	1.00	148	234.61	1.00	1.00	−1.41	0.160	0.06
2020	347	253.72	1.00	1.00	148	234.60	1.00	1.00	−1.46	0.145	0.07
2021	348	254.79	2.00	1.00	148	233.72	1.00	1.00	−1.60	0.110	0.07
2022	351	256.24	2.00	2.00	148	235.20	1.00	1.00	−1.59	0.112	0.07
2023	353	256.94	2.00	1.50	147	235.03	1.00	1.00	−1.65	0.100	0.07
2024	352	257.54	2.00	1.00	150	237.32	1.00	1.00	−1.53	0.127	0.07

*n* – sample; *Me* – median; *IQR* – interquartile range; *Z* – value of the test statistic; *p* – statistical significance; *r* – effect size index; PHC – primary health care.

### 3.5 Implementation of public health tasks in relation to the possession of a Municipal Development Strategy

The analysis revealed a statistically significant correlation between the existence of a Municipal Development Strategy for the years 2019–2024, or part of that timeframe, and the implementation of tasks in the scope of PH (*χ*^2^ = 10.16; *p* = 0.006; Vc = 0.13). Municipalities that had a strategy covering part of this period implemented PH-related tasks much more frequently (77.5%) compared to those without such a strategy (59.3%) (Table 7).

**Table 7:** Implementation of public health tasks in relation to the existence of a Municipal Development Strategy encompassing 2019–2024 or part of this timeframe.

Implementation of public health tasks		Variable			Total	*χ*^2^ (2)	*p*	Vc
		Lack of strategy	Strategy covering the entire period	Strategy covering part of the period				
Yes	*N*	67	235	117	419	10.16	0.006	0.13
	%	59.3	69.9	77.5	69.8			
	Residual	−2.71	0.06	2.37				
No	*N*	46	101	34	181			
	%	40.7	30.1	22.5	30.2			
	Residual	2.71	−0.06	−2.37				
Total	*N*	113	336	151	600			
	%	100	100	100	100			

*N* – sample; *χ*^2^ – chi-square test result; *p* – statistical significance; Vc – effect size index (V Cramer).

## 4 Discussion

Local government communities, specifically municipalities, are best positioned to identify the health needs of their residents and should play a vital role in implementing PH initiatives. Effective PH initiatives rely on joint decision-making among local authorities, dynamic resource management, access to adequate funding, clear and achievable goals, and the employment of staff with the appropriate competencies. These elements are essential for the successful implementation of PH measures at the local level (20–22). Specifically, the collaboration and shared decisions among local authorities significantly contribute to addressing the health needs of residents (23).

This study, to the best of our knowledge, is the first to examine both the extent and the differences in how local authorities implement PH tasks in Poland. Previous research has primarily focused on presenting data regarding implemented Health Policy Programmes (HPPs), which are governed by a separate legal framework and represent only a portion of the broader spectrum of interventions carried out by local government units (24–30). Our analysis takes a more holistic approach. It provides a comprehensive evaluation of the activities undertaken by local governments in fulfilling their PH responsibilities from 2019 to 2024. We place particular emphasis on the disparities among different types of municipalities in the implementation of these tasks, as well as the human resources and infrastructure required for the provision of health services.

Between 2019 and 2024, approximately 70% of the surveyed municipalities in Poland implemented PH tasks. The study’s findings indicate that rural municipalities were the least likely to engage in these activities, which included programmes focused on protective vaccinations, screening tests, and addiction prevention. However, no differences were found in the scope of implementation of PH tasks among individual provinces. A similar correlation was reported in the Woźniak–Holecka 2023 study, which showed that urban municipalities were more likely to implement these PH programmes compared to rural ones (25). In this context, special attention should be given to the number of employees handling PH issues. In most municipalities (around 74%), only one employee is responsible for this area. Our observed correlations suggest that having more staff facilitates the implementation of PH tasks. The shortage of qualified personnel remains a considerable challenge and a major factor hindering the implementation of PH initiatives (25).

In Poland, the Ministry of Health coordinates PH tasks while collaborating with public authorities and subordinate units. Additionally, a Public Health Council is appointed to advise the Ministry of Health, and the Council of Ministers may also designate a Government Plenipotentiary for Public Health (1). In our study, we found that in approximately 65% of municipalities, the local government leader, usually the head of the municipality/mayor/city mayor, was identified as the main initiator of PH task implementation. Other entities were mentioned much less frequently. It is worth noting that Polish legislation does not require local governments to appoint a single coordinator responsible for implementing and overseeing PH tasks. In contrast, such an arrangement has been adopted in England, where local authorities are required to appoint a Director of PH (DPH), who acts as a strategic advisor on health issues to local authority representatives. The DPH, typically a physician, expert or specialist in PH, directs activities in three main areas: health promotion, health protection, and the integration of PH into the healthcare system (31–32). This approach represents a significant step towards helping municipalities implement effective, efficient, and evidence-based health prevention and promotion programmes. However, based on the study’s results and the relevant legal provisions in Poland, the absence of a requirement to employ PH specialists at the local level leads to staff shortages, which may hinder the effectiveness and quality of the health interventions implemented (33). Drawing on guidance for local authorities in England, strengthening local public health capacity in Poland could involve establishing a PH expert or coordinator role within local government structures. Combined with regular population health monitoring and reporting and the development of local health plans based on measurable indicators, such measures could support evidence-informed decision-making and improve the effectiveness of local PH actions (31, 32).

Although having a Municipal Development Strategy was optional, most municipalities planned to have one for the 2019–2024 period (or part of that timeframe). This document can be broad, covering the competence areas of individual local government authorities, or it can focus on specific areas, such as healthcare (34). The Municipal Development Strategy helps coordinate and organise local government activities and commits the municipality to meet its goals within a set timeframe. The Polish legislator appears to recognise the importance of this strategy and its positive impact on the functioning of local governments, as evidenced by the recent amendment to the law, which mandates the possession of such a strategy as of 1 July 2026. However, municipalities in areas within the scope of a supra-local development strategy will be exempt from this requirement (10). According to a Swedish study, long-term, integrated health promotion at the local level demands ongoing political commitment, defined objectives and organised structures. The authors emphasise that such conditions are essential for effectively implementing strategies that advance sustainable PH (35). Our findings confirm that municipalities were more likely to perform PH tasks when they possessed such a strategic document.

Regarding healthcare providers, the study reveals a significant correlation between the implementation of PH tasks and the functioning of hospitals in municipalities from 2019 to 2024. However, this correlation was not evident concerning the number of entities providing primary healthcare services. It is worth noting that most municipalities had only one such entity. Given that the provision of health services is a crucial aspect of PH tasks, the presence of a hospital in a municipality plays a vital role in improving residents’ access to these services. This is particularly true for HPP, where a programme implementer is selected through a tender process, ensuring they meet all specified requirements and provide all the health services outlined in the programme. Interestingly, healthcare units were, right after municipal authorities, the second most frequently indicated initiators of PH initiatives. In contrast, the 2023 study on HPP yielded different findings, indicating that residents were viewed as the primary initiators, followed closely by municipal authorities (25).

Nonetheless, it is unquestionable that during the period investigated, the scope and execution of PH responsibilities were markedly influenced by the COVID-19 pandemic, which compelled municipalities to transfer their resources and funds towards combating the pandemic. Despite these challenges, local authorities rose to the occasion and played a pivotal role in safeguarding residents’ health by implementing measures aimed at mitigating or eliminating the threat’s impact at the time (36, 37).

### 4.1 Strengths and limitations

The fact that this study was conducted on a nationwide scope is undoubtedly one of its key strengths. It substantially contributes to the advancement of health policy and offers a foundational basis for subsequent studies in the domain of PH. The collected data provide practical insights that may facilitate decision-making processes at the local level in the future, particularly in areas such as human resources and municipal management. Moreover, the study encompassed all categories of municipalities, allowing for the observation of differences among them and the identification of potential factors that could impact the extent of PH tasks implementation. Although the research tool was a proprietary questionnaire, it was meticulously developed in collaboration with experts and designed to account for the specific attributes of municipalities operating within Poland.

Certain limitations warrant consideration. Though the survey was directed at local government officials, there is a possibility that it may have been completed by other individuals delegated to do so. This introduces a limitation in verifying the survey process, which could potentially influence the results obtained. Furthermore, the broad and often ambiguous definition of PH, along with the numerous legal provisions within Polish legislation, may have led to varying interpretations of PH responsibilities across different municipalities. This variability may have led to the misinterpretation of some survey questions. Another limitation stems from the non-mandatory nature of the questionnaire item addressing the implementation of vaccination, screening, and addiction prevention programmes. As inconsistencies were identified in the responses provided by some municipalities, these cases were excluded from the relevant analyses. As a result, the reported estimates for specific preventive interventions may be subject to under- or overestimation, which may have affected the interpretation of the findings. Moreover, due to the unfavourable outcomes observed in statistically significant findings, it is essential to approach these results with caution.

## 5 Conclusion

Most municipalities in Poland implement PH tasks. However, the scope of implementation of these tasks is considerably higher in urban municipalities compared to rural ones. Factors such as the number of employees responsible for implementing PH tasks, the presence of hospitals within the municipality, and the existence of a Municipal Development Strategy can significantly influence the extent of PH measures undertaken by municipalities.

The study’s findings suggest that rural municipalities may face resource limitations that hinder effective activities aimed at promoting citizens’ health. Therefore, efforts should focus on identifying these issues and disparities, followed by the implementation of suitable measures to support the development of health policies in smaller local governments.

## Data Availability

The raw data supporting the conclusions of this article will be made available by the authors, without undue reservation.
