# Supplementary material for: Implementation of public health tasks by Polish municipalities in 2019–2024: a cross-sectional study
Source: Front Public Health. 2026 Sep 10;14:1932172. doi: 10.3389/fpubh.2026.1932172 (PMC13601248; doi:10.3389/fpubh.2026.1932172)
Supplement: Supplementary file 1 [file Table_1.DOCX]

**Table S1. Number and percentage of completed questionnaires by province (N = 601)**

| **Province** | ***n*** | **%** |
| --- | --- | --- |
| Lower Silesian | 28 | 4.7 |
| Kuyavian-Pomeranian | 38 | 6.3 |
| Lublin | 59 | 9.8 |
| Lubusz | 20 | 3.3 |
| Łódź | 40 | 6.7 |
| Lesser Poland | 46 | 7.7 |
| Masovian | 62 | 10.3 |
| Opole | 14 | 2.3 |
| Subcarpathian | 50 | 8.3 |
| Podlaskie | 29 | 4.8 |
| Pomeranian | 30 | 5.0 |
| Silesia | 47 | 7.8 |
| Świętokrzyskie | 31 | 5.2 |
| Warmian-Masurian | 27 | 4.5 |
| Greater Poland | 55 | 9.2 |
| West Pomeranian | 25 | 4.2 |

n – sample

**Table S2. Initiators of public health tasks (among municipalities that implemented these tasks)**

| **Initiators** | **Primary initiator*** | | **All initiators**** |
| --- | --- | --- | --- |
|  | ***n*** | **%** | ***n*** |
| Head of Municipality/Mayor/City Mayor | 272 | 64.8 | 328 |
| healthcare units | 35 | 8.3 | 88 |
| county self-government | 23 | 5.5 | 45 |
| others | 22 | 5.2 | 38 |
| members of the municipal council | 20 | 4.8 | 69 |
| provincial self-government | 13 | 3.1 | 29 |
| educational establishments | 11 | 2.6 | 67 |
| non-governmental organisations | 10 | 2.4 | 61 |
| committee responsible for health protection | 7 | 1.7 | 24 |
| State Sanitary Inspectorate | 5 | 1.2 | 12 |
| commercial companies, e.g. pharmaceutical companies | 2 | 0.5 | 11 |
| residents of the municipality | 0 | 0 | 42 |

* single-choice question (N = 420)

* multiple-choice question

**Table S3. Implementation of public health tasks by province type in the years 2019–2024**

| **Province** | **Variable** | | | | | | **χ² (15)** | ***p*** | **Vc** |
| --- | --- | --- | --- | --- | --- | --- | --- | --- | --- |
|  | Yes | | | No | | |  |  |  |
|  | *n* | *%* | residual | *n* | *%* | residual |  |  |  |
| Lower Silesian | 20 | 71.4 | 0.20 | 8 | 28.6 | -0.20 | 24.40 | 0.059 | 0.20 |
| Kuyavian-Pomeranian | 34 | 89.5 | 2.70 | 4 | 10.5 | -2.70 |  |  |  |
| Lublin | 39 | 66.1 | -0.70 | 20 | 33.9 | 0.70 |  |  |  |
| Lubusz | 14 | 70.0 | 0.00 | 6 | 30.0 | 0.00 |  |  |  |
| Łódź | 28 | 70.0 | 0.00 | 12 | 30.0 | 0.00 |  |  |  |
| Lesser Poland | 36 | 78.3 | 1.30 | 10 | 21.7 | -1.30 |  |  |  |
| Masovian | 40 | 64.5 | -1.00 | 22 | 35.5 | 1.00 |  |  |  |
| Opole | 8 | 57.1 | -1.10 | 6 | 42.9 | 1.10 |  |  |  |
| Subcarpathian | 29 | 58.0 | -1.90 | 21 | 42.0 | 1.90 |  |  |  |
| Podlaskie | 20 | 69.0 | -0.10 | 9 | 31.0 | 0.10 |  |  |  |
| Pomeranian | 22 | 73.3 | 0.40 | 8 | 26.7 | -0.40 |  |  |  |
| Silesia | 40 | 85.1 | 2.40 | 7 | 14.9 | -2.40 |  |  |  |
| Świętokrzyskie | 17 | 54.8 | -1.90 | 14 | 45.2 | 1.90 |  |  |  |
| Warmian-Masurian | 16 | 59.3 | -1.20 | 11 | 40.7 | 1.20 |  |  |  |
| Greater Poland | 39 | 70.9 | 0.20 | 16 | 29.1 | -0.20 |  |  |  |
| West Pomeranian | 18 | 72.0 | 0.20 | 7 | 28.0 | -0.20 |  |  |  |
| **Total** | 420 | 69.9 |  | 181 | 30.1 |  |  |  |  |

*n* - sample; χ² – chi-square test result; *p* – statistical significance; Vc – effect size index (V Cramer)

**Table S4. Number of employees engaged in public health issues by province type in 2019–2024**

| **Province** | ***n*** | **Mean**  **rank** | ***Me*** | ***IQR*** | ***H* (15)** | ***p*** | **η^2^** |
| --- | --- | --- | --- | --- | --- | --- | --- |
| Lower Silesian | 28 | 294.98 | 2.00 | 0.00 | 19.17 | 0.206 | 0.01 |
| Kuyavian-Pomeranian | 38 | 340.03 | 2.00 | 1.00 |  |  |  |
| Lublin | 59 | 322.17 | 2.00 | 0.00 |  |  |  |
| Lubusz | 20 | 277.38 | 2.00 | 0.00 |  |  |  |
| Łódź | 40 | 295.04 | 2.00 | 0.00 |  |  |  |
| Lesser Poland | 46 | 321.07 | 2.00 | 0.00 |  |  |  |
| Masovian | 62 | 315.83 | 2.00 | 0.00 |  |  |  |
| Opole | 14 | 307.64 | 2.00 | 0.00 |  |  |  |
| Subcarpathian | 50 | 287.72 | 2.00 | 0.00 |  |  |  |
| Podlaskie | 29 | 285.91 | 2.00 | 0.00 |  |  |  |
| Pomeranian | 30 | 325.50 | 2.00 | 0.00 |  |  |  |
| Silesia | 47 | 281.85 | 2.00 | 0.00 |  |  |  |
| Świętokrzyskie | 31 | 238.29 | 2.00 | 1.00 |  |  |  |
| Warmian-Masurian | 27 | 292.65 | 2.00 | 0.00 |  |  |  |
| Greater Poland | 55 | 282.16 | 2.00 | 0.00 |  |  |  |
| West Pomeranian | 25 | 328.36 | 2.00 | 0.00 |  |  |  |

*n* – sample; *Me* – median; *IQR* – interquartile range; *H* – result of the Kruskal-Wallis test; *p* – statistical significance; η² – effect size index (eta squared)

**Table S5. Implementation of public health tasks based on the number of staff engaged in public health issues in 2019–2024**

| **Dependent variable** | **Yes (*n* = 420)** | | | **No (*n* = 181)** | | | ***Z*** | ***p*** | ***r*** |
| --- | --- | --- | --- | --- | --- | --- | --- | --- | --- |
|  | mean rank | *Me* | *IQR* | mean rank | *Me* | *IQR* |  |  |  |
| Number of employees | 325.36 | 2.00 | 0.00 | 244.48 | 2.00 | 0.00 | -6.80 | <0.001 | 0.28 |

*n* – sample; *Me* – median; *IQR* – interquartile range; *Z* – value of test statistic; *p* – statistical significance; *r* – effect size index

**Appendix 1. Survey questionnaire**

(in editable form, designed in MS Word)

**The survey questionnaire consists of four sections.**

**The first section** provides a space for entering the municipality identifier, based on the National Official Register of Territorial Division (TERYT). This identifier will be anonymised and not included in any published materials.

**The second section** comprises five questions. The questions are related to general information about the municipality's management and social infrastructure.

**Section three** features eight questions. They relate to public health, including information on funding sources for related activities and obstacles faced during planning, implementation, executing, and reporting of public health tasks.

**Section four** has two questions plus one additional question. The questions cover areas regarding disease prevention and opinions on key elements pertinent to public health tasks.

Data obtained from the study will be used to implement scientific tasks.

It takes a maximum of 25 minutes to complete the questionnaire.

Section I

**TERYT of the Municipality**

*Data regarding the municipality will be anonymised and not included in any published materials.*

Section II

1. Type of municipality:
   - urban
   - rural
   - urban-rural
2. Did the municipality have a Municipal Development Strategy covering 2019–2024 or part of this timeframe?
   - no (if no, please proceed to question 3)
   - yes, it had a strategy covering the entire period (if yes, please proceed to question 2.1)
   - yes, it had a strategy covering part of that period (if yes, please proceed to question 2.1)*

**Please specify the years*

2.1 Did this strategy include objectives related to the implementation of public health tasks?
- yes
- no

3. Did a hospital operate in the municipality between 2019 and 2024?
   - yes
   - no
4. Please specify the number of Primary Health Care (PHC) providers operating in the municipality between 2019 and 2024:

| **Year** | **2019** | **2020** | **2021** | **2022** | **2023** | **2024** |
| --- | --- | --- | --- | --- | --- | --- |
| Number of entities in a given year | …… | …… | …… | …… | …… | …… |
| The municipality has no information on the number of PHC entities |  |  |  |  |  |  |

5. What percentage of the municipal budget between 2019 and 2024 was allocated to public health tasks? (optional question)

*Public health is understood as the tasks defined in Article 2 of the Public Health Act of 11 September 2015, such as monitoring and evaluation of the health status of the population, health education, health promotion, and disease prevention, among others.*

| **Year** | **2019** | **2020** | **2021** | **2022** | **2023** | **2024** |
| --- | --- | --- | --- | --- | --- | --- |
| % | …… | …… | …… | …… | …… | …… |

Section III

6. Did the municipality obtain funds for public health tasks from external national sources in the period 2019–2024?
   - yes (if yes, please proceed to question 6.1)
   - no (if no, please proceed to question 6.2)
   6.1. Please provide these sources:

Multiple answers possible

- funds from other local authorities
- funds from national public benefit organisations
- funds from commercial companies, e.g., pharmaceutical companies
- funds from the National Health Fund
- funds from the Medical Fund
- funds from government administration*

**Please specify the government administration authority*

- other funds (which?)**

***Please provide other national sources*

6.2. Did the municipality apply for funding from external national sources for public health tasks between 2019 and 2024?
- yes
- no

7. Did the municipality obtain funding for public health tasks from foreign sources between 2019 and 2024?
   - yes (if yes, please proceed to question 7.1)
   - no (if no, please proceed to question 7.2)
   7.1. Please provide these sources:

Multiple answers possible

- European Union
- Norwegian Funds
- other foreign funds (which?)*

**Please provide other foreign sources*

7.2. Did the municipality apply for funding from foreign sources for public health tasks between 2019 and 2024?
- yes
- no

8. Who was/were the initiator(s) of public health tasks?

Multiple answers possible

- the municipality did not implement public health tasks in the period 2019–2024 (please proceed to question 10)
- members of the municipal council
- Head of Municipality/Mayor/City Mayor
- county self-government
- provincial self-government
- commercial companies, e.g., pharmaceutical companies
- non-governmental organisations
- healthcare units
- educational establishments
- residents of the municipality
- State Sanitary Inspectorate
- committee responsible for health protection
- other (which?)*

**Please provide other initiators*

9. Who was the primary initiator of the public health tasks?

*Please indicate one entity from those listed in question 8*

10. What, in your opinion, hinders the municipality's implementation of its public health tasks?

Multiple answers possible

- lack of or inconsistent legislation
- limited financial capacity of the municipality
- lack of qualified staff
- problems with finding a programme implementer
- difficulties in enrolling participants in programmes
- burden of other municipal tasks
- other factors (which?)*

**Please indicate other factors*

11. What, in your opinion, is the key factor hindering the implementation of public health tasks?

*Please indicate one factor among those specified in question 10*

12. Please indicate the current number of municipal employees engaged in public health issues:
    - 0
    - 1
    - 2
    - 3
    - 4
    - if more (how many?)*

**Please indicate the number of employees*

13. Did you encounter any problems when reporting on public health tasks?
    - yes (if yes, please proceed to questions 13.1. and 13.2.)
    - no

13.1 What are the problems?

Multiple answers possible

- too many fields to be filled in
- incomprehensible explanations of the fields for filling in information on the implemented tasks
- lack/insufficient training on how to correctly fill in reports
- insufficient number of employees
- time-consuming to complete the report
- other (which?)*

**Please list other barriers/problems*

13.2 Please indicate solutions which, in your opinion, could make it easier for local government units to report on public health tasks (optional question)

**Please provide solutions*

Section IV

14. Please tick the problems that arose during the implementation of tasks related to protective vaccinations, screening tests, and addiction prevention.

In each column listing interventions, you may select more than one issue indicated in the rows.

| **Problems related to the implementation of tasks** | **Area of prevention** | | |
| --- | --- | --- | --- |
|  | Preventive vaccinations | Screening tests | Addiction prevention |
| The municipality did not implement prevention activities in the areas indicated between 2019 and 2024 |  |  |  |
| Low enrolment of participants in the programme |  |  |  |
| Limited financial capacity of the municipality |  |  |  |
| Problems in selecting an implementer |  |  |  |
| Difficulties in cooperating with the implementer |  |  |  |
| Overly formalised procedures |  |  |  |
| The impact of the COVID-19 pandemic |  |  |  |
| Problems with coordinating activities in the municipality |  |  |  |
| Difficulties accessing vaccines |  |  |  |
| Increase in vaccine prices |  |  |  |
| Other (which?) please proceed to questions 14.1* |  |  |  |
| No problems arose during the implementation of the tasks |  |  |  |

14.1 Please provide other problems that arose during the implementation of tasks.

**Please provide other problems*

15. To what extent do you agree with the following statements?

| **Statement** | **I strongly disagree** | **I disagree** | **I neither agree nor disagree** | **I agree** | **I strongly agree** |
| --- | --- | --- | --- | --- | --- |
| Municipalities in Poland are subject to an excessive number of regulations governing public health. |  |  |  |  |  |
| The numerous legal frameworks regulating municipal public health activities hinder the implementation of preventive programmes. |  |  |  |  |  |
| The potential for securing funding motivates municipalities to act in disease prevention. |  |  |  |  |  |
| Reporting on the effects of disease prevention measures is a crucial component. |  |  |  |  |  |
| The number of individuals participating in a particular disease prevention activity is a good indicator of its effectiveness. |  |  |  |  |  |
| The need to obtain an opinion from AOTMiT (Agency for Health Technology Assessment and Tariff System) discourages the development of health policy programmes. |  |  |  |  |  |
| The President of AOTMiT's recommendations on medical technologies, actions, and conditions for implementing health policy programmes for a specific disease or health issue facilitate the implementation of disease prevention programmes by municipalities. |  |  |  |  |  |

16. If you have any comments or feedback regarding the implementation of public health tasks or this survey, please provide them below (optional question).
